# Minimally invasive versus traditional open transforaminal lumbar interbody fusion for the treatment of low-grade degenerative spondylolisthesis: a retrospective study

**DOI:** 10.1038/s41598-020-78984-x

**Published:** 2020-12-14

**Authors:** Rongqing Qin, Tong Wu, Hongpeng Liu, Bing Zhou, Pin Zhou, Xing Zhang

**Affiliations:** 1 Department of Spinal Surgery, Gaoyou Hospital Affiliated Soochow University, Gaoyou, 225600 Jiangsu China; 2 Department of Orthopedics, Gaoyou People’s Hospital, Gaoyou, 225600 Jiangsu China; 3 Department of Spinal Surgery, Affiliated Hospital of Nantong University, Nantong, 226001 Jiangsu China; 4 Department of Orthopedics, Gaoyou Hospital of Integrated Traditional Chinese and Western Medicine, Gaoyou, 225600 Jiangsu China

**Keywords:** Orthopaedics, Neurological disorders, Spinal cord injury

## Abstract

This was a retrospective study. We aimed to compare the clinical efficacy and safety between minimally invasive and traditional open transforaminal lumbar interbody fusion in the treatment of low-grade lumbar degenerative spondylolisthesis (LDS). 81 patients with LDS grades 1 and 2 treated in our spinal department from January 2014 to July 2016 were retrospectively analyzed. The MIS-TLIF group included 23 males and 11 females, while the TO-TLIF group included 29 males and 18 females. Follow-up points were set at 7 days, 3 months, 6 months, 12 months postoperatively and the last follow-up. Various clinical and radiological indicators were used to evaluate and compare the efficacy and safety between the two procedures. 8 cases (3 in the MIS-TLIF group and 5 in the TO-TLIF group) were loss of follow-up after discharge. And the remaining 73 patients were followed up for at least 2 years. No statistically significant difference was observed in the terms of age, sex, BMI, slippage grade, and surgical segments. The MIS-TLIF group had a longer operation and fluoroscopy time compared with the TO-TLIF group. But the MIS-TLIF group was associated with less blood loss, ambulation time, hospital stay, and time of return to work. In each group, significant improvement were observed in BP-VAS, ODI and vertebral slip ratio at any time-point of follow-up when compared with the preoperative condition. When the time-point of follow-up was less than 1 year, the MIS-TLIF group had significant advantages in the BP-VAS and ODI compared with TO-TLIF group. But no significant difference was observed in the BP-VAS and ODI at either 12 month follow-up or the last follow-up. Besides, no statistical difference was detected in vertebral slip ratio at any time-point of follow-up between the two groups. Successful intervertebral bone fusion was found in all patients and no significant difference was found in the incidence of total complications. Thus, we considered that MIS-TLIF and TO-TLIF both achieve satisfactory clinical efficacy in the treatment of low-grade single-segment LDS. But MIS-TLIF appears to be a more efficacious and safe technique with reduced tissue damage, less blood loss and quicker recovery.

## Introduction

Most patients with low-grade degenerative lumbar spondylolisthesis (DLS) have no obvious symptoms, but some patients may have lower back pain with or without lower limb radiation pain. Generally, conservative treatment can relieve symptoms. But for patients who fail conservative treatment, surgical treatment has been identified as having more advantages^1^. Many surgical techniques have been used to treat symptomatic DLS. And the ultimate therapeutic goal is to stabilize the spine segment and decompress neural elements^2^. In 1982, Harms first described transforaminal lumbar interbody fusion (TLIF)^3^, which was later popular for its effective treatment of degenerative lumbar disease^4^. Compared with posterior lumbar interbody fusion (PLIF), TLIF technique reduced traction on the dura sac and nerve roots, reduced iatrogenic nerve-related complications, and preserved the structural integrity of the posterior column^5,6^. However, many subsequent studies had reported the harmful effects of this traditional open TLIF technique (TO-TLIF), such as excessive paraspinal muscle dissection and intraoperative bleeding^7,8^. There were even reports in the literature that excessive paraspinal muscle dissection might cause muscle denervation, ischemic necrosis and blood remodeling disorders, leading to muscle atrophy and scar formation^9^. With the innovation of concepts and the development of surgical instruments and optical equipment, minimally invasive spinal surgery is the general trend. In 2002, Foley first reported the minimally invasive TLIF technique (MIS-TLIF)^10^. Due to the smaller incision, less iatrogenic soft tissue damage, and faster functional recovery, MIS-TLIF technique is becoming more and more popular^11–13^. However, there is still no high-quality evidence to prove which procedure is superior in the treatment of low-grade single-segment DLS. And this study was performed with the purpose of estimating and comparing the clinical efficacy and safety between MIS-TLIF and TO-TLIF.

## Materials and methods

### Patient population

It was a observational study. After approval by the Institutional Review Board of Gaoyou Hospital Affiliated Soochow University, we retrospectively enrolled a total of 81 patients (34 cases in MIS-TLIF group and 47 cases in TO-TLIF group) who suffered persistent mechanical lower back pain with or without lower limb radiation pain from January 2014 to July 2016. Informed consent was obtained from all subjects. We made the surgical decision for MIS versus TO-TLIF under the precondition of patients’ general condition and preference. 34 patients who experienced MIS-TLIF and pereutaneous pediele screw fixation were set as the MIS-TLIF group, while 47 cases with traditional open TLIF and pedicle screw fixation were set as the TO-TLIF group.

### Inclusive and exclusive criteria

Inclusive criteria: 1) The age of patients was ranged from 50 to 80 years old; 2) Single-segment degenerative spondylolisthesis grade 1 or 2, which confirmed by imaging examination; 3) Persistent mechanical lower back pain with or without lower limb radiation pain; 4) Imaging characteristics were in accordance with clinical symptoms; 5) Regular conservative treatment had no obvious efficacy over 3 months, or recurrent attacks of the symptom; 6) The duration of follow-up was at least 2 years. Exclusive criteria: 1) Two or more segments of lumbar spondylolisthesis; 2) lumbar spondylolisthesis grade 3 or grade 4; 3) Patient had a history of lumbar spine surgery; 4) Patients with infectious diseases, metabolic bone diseases, spinal trauma, deformities or tumors; 5) Patients with severe medical complications who cannot tolerate surgery.

### Surgical procedures

#### TO-TLIF procedure

TO-TLIF technology used the Harms classic method^14^. After successful general anesthesia, the patient was in a prone position with chest and hip pads to avoid pressure on the abdomen. After taking the posterior median incision (with the diseased vertebrae as the center), we cut the skin, subcutaneous tissue and fascia in turn, then peeled off the paraspinous muscles on both sides along the spinous process to expose the posterior anatomical structure. The pedicle screws were placed after satisfactory positioning. According to the patient’s symptom and spinal canal anatomy, part or all of the upper and lower articular processes were removed, and part of the lamina was removed to expand the central spinal canal and nerve root canal. After decompression thoroughly of the dural sac, walking roots and traveling roots, the nucleus pulposus was removed with forceps and the endplate cartilage was scraped off using curette. We first lifted and reduced the spondylolisthesis, then flushed the intervertebral space, implanted autogenous bone and bone morphogenetic proteins (BMP). After the mold trial, a suitable PEEK cage was placed in the center of the intervertebral space through the Kambin triangle area. The nuts were loosed side by side, the intervertebral space was compressed and finally the nuts were tightened and fixed.

#### MIS-TLIF procedure

After successful general anesthesia, the patient was placed in a prone position with chest and hip pads to avoid abdominal compression. Before operation, the C-arm machine was used to fluoroscopically locate the surgical segment and mark the pedicle. After routine disinfection and towel laying, we made a longitudinal incision of about 2.5 cm at the marked position (Wiltse approach), cut the skin, subcutaneous tissue and deep fascia in sequence, and then bluntly separated the gap between the multifidus muscle and the longest muscle to reach the articular process, and revealed the position of the nail, inserted the cannula in stages, and installed the Quadrant channel device (Sofamor, USA). After fluoroscopy again, the operation segment was correct, and part or all of the upper and lower articular processes were selected to be removed according to the patient's symptoms and spinal canal anatomy, and part of the lamina was removed to enlarge the spinal canal. We exposed the dural sac, walk and travel nerve roots and release the decompression. The nucleus pulposus was removed from the degenerated nucleus, the endplate cartilage was scraped off, and the autogenous bone and BMP was implanted and beaten tightly. After successful mold trial, a suitable PEEK cage was placed into the center of the intervertebral space through the Kambin triangle area. The pedicle screw was placed in this incision first, then the two pedicle screws were inserted percutaneously on the opposite side, and the fixation rod was inserted with the assistance of the navigation device. The degree of pull reduction is determined according to the degree of slippage. During pull reduction, the whole process is performed under fluoroscopy, and finally the pressure is fixed. Look again to confirm that the screws and cage are in place. After the operation, physiological saline was flushed out, and after removing active bleeding, a latex tube was placed on the decompression side, and the incision was sutured layer by layer.

### Efficacy evaluation

We used back pain visual analogue scale (BP-VAS) score and Oswestry disability index (ODI) score at 7 days, 3 months, 6 months, 12 months postoperatively and at the last follow-up to evaluate the clinical efficacy. And the excellent and good rate based on the modified Macnab criteria was uesd for patient satisfaction evaluation (Excellent: preoperative symptoms disappear thoroughly, restore the normal life and work; Good: slightly restricted activity, occasional non-radicular pain, and no impact on the life and work; Fair: clinical symptoms are relieved, the activity is limited, and the normal life and work are affected; Poor: no difference in symptoms before and after surgery, even aggravation). We also recorded the operation time, intraoperative fluoroscopy time, intraoperative blood loss, postoperative drainage volume, time of bed rest, duration of hospitalization, time to return to work, and complications with subsequent remedy. Besides, radiological evaluation was conducted via the vertebral slip ratio, as well as the fusion rate which based on the Bridwell standard^15^.

### Statistical analysis

All statistical analysis were performed via SPSS 21.0 software (IBM Corp., Armonk, NY). Continuous variables were expressed as mean ± standard deviation. The independent sample t test was used for the comparison of continuous variables between groups, and the chi-square test was used for the comparison of binary variables between groups. Wilcoxon rank sum test was used to compare rank variables between groups. And paired t test was used to compare the continuous variables within the group. *P* value of less than 0.05 was considered statistically significant.

### Ethics approval

We confirm that all methods were carried out in accordance with relevant guidelines and regulations.

## Results

**Figure 1:**
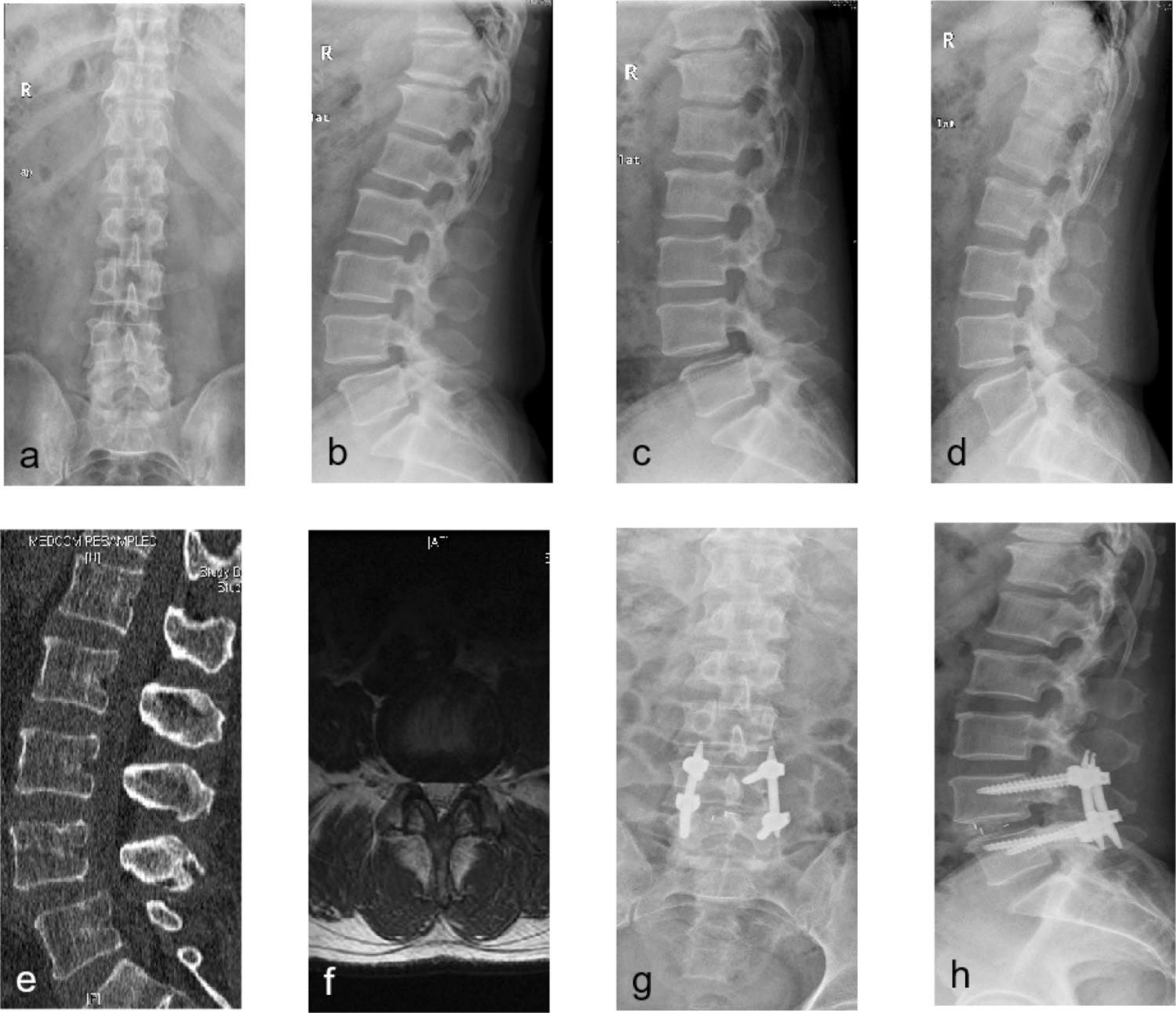
A 61 years old male patient who suffered from low-back pain for 16 months prior to admission underwent MIS-TLIF procedure. (**a**,**b**) Antero-posterior and lateral plain radiography before operation; (**c**,**d**) preoperative flexion and extension plain radiography indicated patient with LDS grade 1; (**e**,**f**) preoperative sagittal CT scan and axial T2-weighted MR image; (**g**,**h**) antero-posterior and lateral plain radiography after surgery.

**Figure 2:**
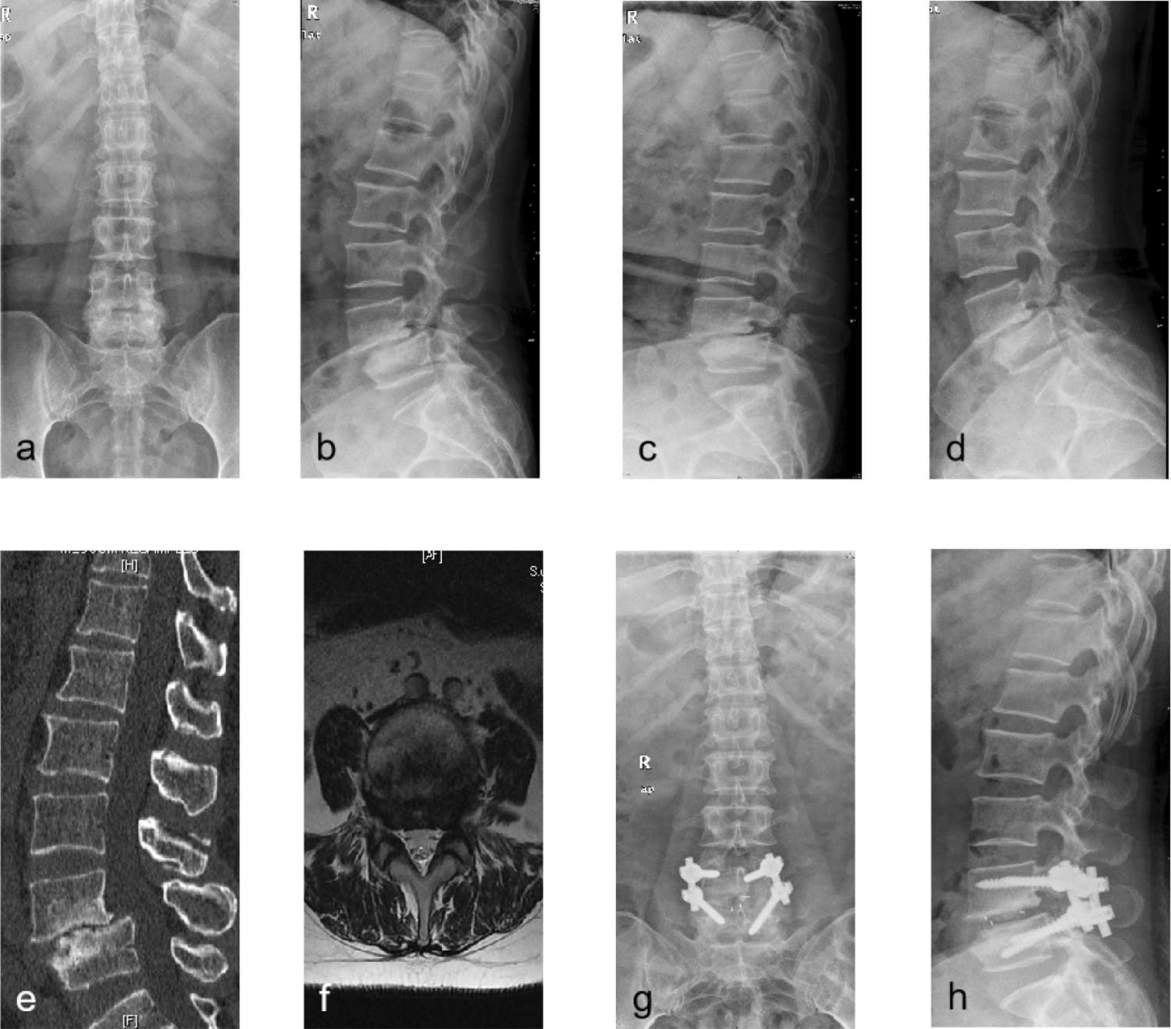
A 57 years old female patient who suffered from low-back pain with radicular pain of left lower extremity for 7 months prior to admission underwent TO-TLIF procedure. (**a**,**b**) Preoperative antero-posterior and lateral plain radiography; (**c**,**d**) preoperative flexion and extension plain radiography indicated patient with LDS grade 1; (**e**,**f**) preoperative sagittal CT scan and axial T2-weighted MR image; (**g**,**h**) antero-posterior and lateral plain radiography after surgery.

The MIS-TLIF group included 23 males and 11 females, with an average age of 66.09 ± 8.19 years. Among them, 21 cases were degenerative spondylolisthesis grade 1, while 13 cases were grade 2. The TO-TLIF group included 29 males and 18 females, with an average age of 65.81 ± 8.51 years. And 32 cases were degenerative spondylolisthesis grade 1, while 15 cases were grade 2. No significant difference was observed in terms of age, gender, body mass index (BMI), slippage grade, surgical segments and pre-admission disease course between the two groups (*P* > 0.05, Table 1). 8 patients (3 cases in MIS-TLIF group and 5 cases in TO-TLIF group) were lost to follow-up after discharge. And the remaining 31 patients in the MIS-TLIF group had an average follow-up of 31.15 ± 5.56 months, while 42 patients in the TO-TLIF group followed up for a mean duration of 29.91 ± 4.63 months. Typical cases experienced MIS/Open-TLIF were illustrated in Figs. 1 and 2 respectively.

**Table 1:** Baseline characteristics of the patients.

	MIS-TLIF group (n = 34)	TO-TLIFgroup (n = 47)	*P* value
Age (years)	66.09 ± 8.19	65.81 ± 8.51	0.704
Male/female	23/11	29/18	0.582
BMI	25.09 ± 3.27	24.40 ± 2.91	0.325
Slippage grade (1/2)	21/13	32/15	0.556
Surgical segment			0.653
L3–L4	11	15	
L4–L5	16	21	
L5–S1	7	11	
Pre-admission disease course (month)	45.82 ± 12.02	46.51 ± 12.53	0.805

### Various indicators during perioperative period

The operation time and intraoperative fluoroscopy time in the MIS-TLIF group were longer than those in the TO-TLIF group, and the difference between the two groups was statistically significant (*P* < 0.05, Table 2). However, the intraoperative blood loss, postoperative drainage volume, time of bed rest, and hospital stay in the MIS-TLIF group were significantly less than those in the TO-TLIF group, and the difference was statistically significant (*P* < 0.05, Table 2).

**Table 2:** Comparation of surgical indicators between the two groups (*P < 0.05).

	MIS-TLIF (n = 34)	TO-TLIF (n = 47)
Operation time (min)	143.94 ± 11.59	113.70 ± 9.44*
Intraoperative blood loss (ml)	170.56 ± 11.98	261.70 ± 12.33*
Intraoperative fluoroscopy time (s)	39.92 ± 3.34	17.89 ± 1.33*
Postoperative drainage volume (ml)	104.21 ± 8.99	157.72 ± 10.46*
Time of bed rest (day)	3.32 ± 0.42	10.65 ± 2.19*
Hospital stay (day)	10.26 ± 1.35	13.41 ± 1.52*

### Patient-reported clinical indicators

The time to return to work was significantly shorter in the MIS-TLIF group when compared with TO-TLIF group, and the difference was statistically significant (*P* < 0.05, Table 3).

**Table 3:** Comparation of patient-reported outcome measures.

Time to return to work (month)	MIS-TLIF (n = 31)		TO-TLIF (n = 42)	
	2.31 ± 0.48		3.52 ± 0.91*	
	BP-VAS (0–10)	ODI (%)	BP-VAS (0–10)	ODI (%)
Preoperative scores	7.73 ± 0.53	42.34 ± 4.61	7.49 ± 0.68	42.16 ± 5.95
7 days postoperatively	3.38 ± 0.27‡	25.45 ± 3.12‡	4.75 ± 0.62‡*	31.76 ± 2.79‡*
3 months postoperatively	3.12 ± 0.26‡	21.93 ± 3.58‡	4.23 ± 0.58‡*	27.52 ± 3.34‡*
6 months postoperatively	2.33 ± 0.21‡	17.27 ± 2.47‡	3.51 ± 0.39‡*	21.35 ± 3.53‡*
12 months postoperatively	1.94 ± 0.35‡	15.88 ± 2.07‡	2.01 ± 0.53‡	16.13 ± 3.15‡
Last follow-up	1.26 ± 0.27‡	14.71 ± 1.21‡	1.39 ± 0.42‡	15.23 ± 1.97‡

*P < 0.05, comparation of indicators at each time point between the two groups.

^‡^*P* < 0.05, Scores at each time point postoperatively versus preoperative score. BP-VAS: low-back pain VAS scores.

**Figure 3:**
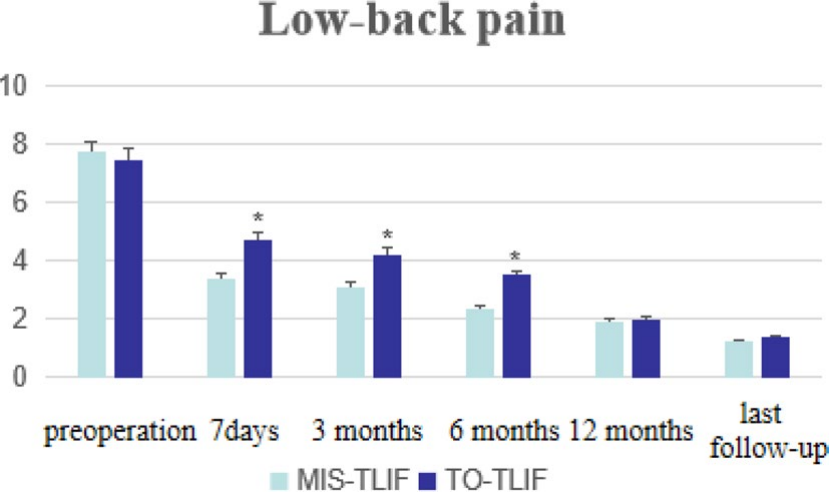
Histograms for low-back pain VAS scores (*P < 0.05, comparation between the two groups).

**Figure 4:**
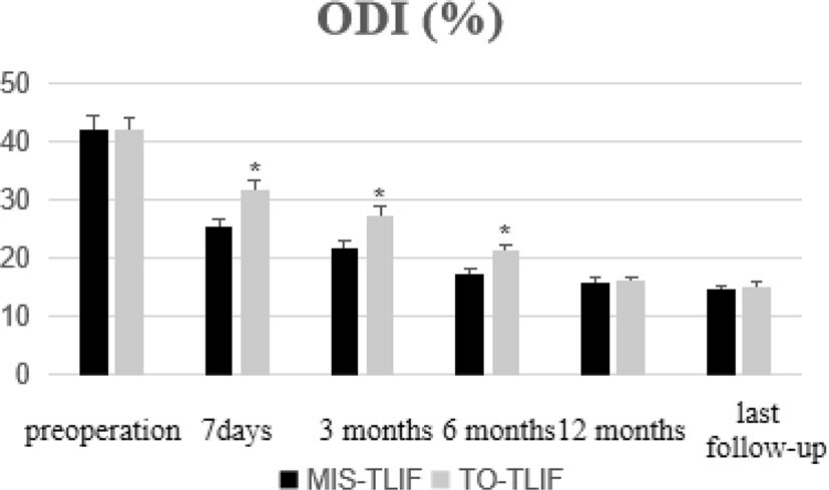
Histograms for ODI scores (*P < 0.05, comparation between the two groups).

No statistically significant differences were observed in the preoperative BP-VAS and ODI scores between the two groups (*P* > 0.05). In each group, significant improvement were observed in BP-VAS and ODI scores at any time-point of follow-up when compared with the preoperative condition (*P* < 0.05). When the time-point of follow-up was less than 1 year (7 days, 3 months and 6 months after surgery), the MIS-TLIF group was superior in the BP-VAS and ODI scores compared with the TO-TLIF group, and the differences were statistically significant (*P* < 0.05). But no statistical differences were observed in both BP-VAS and ODI scores at the time-point of 12 months postoperatively and at the last follow-up between the two groups (*P* > 0.05). See Table 3, Figs. 3 and 4 for details.

In the MIS-TLIF group, the clinical efficacy was excellent for 21 cases, good for 7 cases, and fair for 3cases with an excellent and good rate of 90.3%. While in the TO-TLIF group, the efficacy was excellent for 26 cases, good for 11 cases, and fair for 5 cases with an excellent and good rate of 88.1%. No statistically significant difference was observed in the excellent and good rate at the last follow-up between the two groups (*P* > 0.05).

### *V*ertebral slip ratio

**Figure 5:**
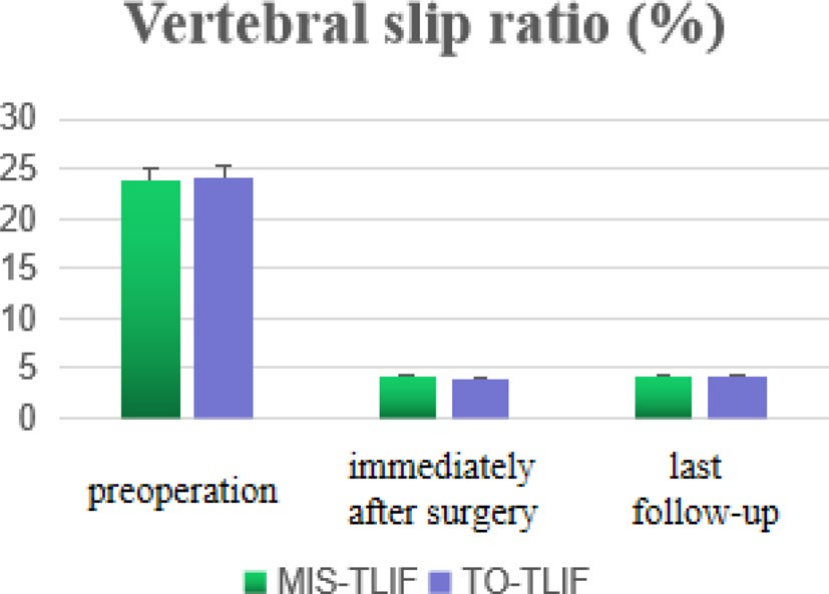
Histograms for vertebral slip ratio between MIS-TLIF and TO-TLIF groups.

In each group, statistically significant improvement in vertebral slip ratio was observed at time-point of immediately after surgery and last follow-up when comparing with preoperative status (*P* < 0.05). And no significant difference was exist in vertebral slip ratio between the status of last follow-up and immediately after surgery in each group (*P* > 0.05). Moreover, no statistical difference was detected in vertebral slip ratio at any time-point of follow-up between the MIS-TLIF group and TO-TLIF group (*P* > 0.05, Table 4 and Fig. 5).

**Table 4:** Comparation of vertebral slip ratio between the two groups.

Vertebral slip ratio (%)	MIS-TLIF (n = 34)	TO-TLIF (n = 47)
Preoperatively	23.87 ± 8.45	24.12 ± 9.33§
Immediately after surgery	4.09 ± 2.36*	3.97 ± 2.51*§
	MIS-TLIF (n = 31)	TO-TLIF (n = 42)
Last follow-up	4.16 ± 2.13*‡	4.05 ± 2.27*‡§

*P < 0.05, vertebral slip ratio at each time point postoperatively versus preoperative score in each group.

^‡^*P* > 0.05, slip ratio at last follow-up versus immediately after surgery in each group.

^§^*P* > 0.05, comparation of vertebral slip ratio at each time point between the two groups.

### Complications

No statistically significant difference was found in the incidence of total complications (*P* > 0.05, Table 5). One case of superficial incision infection occurred in the MIS-TLIF group, and two cases occurred in the TO-TLIF group, which were all cured by intensive dressing and anti-infection treatment. No deep infection occurred in both groups. In the TO-TLIF group, there was a case of cerebrospinal fluid leakage after operation. The patient was placed in a head-down position and treated by fluid replacement and preventing infection. After delayed extubation and intermittent clamping treatment, it improved within 1 week after operation and no obvious neurological deficit occurred. Besides, no complications such as screw loosening and cage displacement were found in both groups. One case in the MIS-TLIF group felt numbness of left lower extremity postoperatively and recovered completely after 4 weeks of conservative treatment. No aggravation of the preoperative medical comorbidities was exist in either MIS-TLIF group or TO-TLIF group.

**Table 5:** Comparation of surgical-related complications between the two groups.

	Superficial incision infection	Numbness of left lower extremity	Cerebrospinal fluid leakage	Total (n)
MIS-TLIF (n = 31)	1	1	0	2 (6.45%)
TO-TLIF (n = 42)	2	0	1	3 (7.14%)

### Fusion and reoperation rate

Complete interbody fusion was detected in both groups. None of patients received reoperation during the period of follow-up.

## Discussion

In the last few decades, the advent of minimally invasive techniques in the spinal surgery led to the logical progression of TO-TLIF to MIS-TLIF^8^. The MIS-TLIF technique has been praised due to its advantages of smaller incision, less bleeding, and faster recovery compared with TO-TLIF^16^. However, there is still a lack of powerful evidence to prove which procedure has a better clinical efficacy in treating symptomatic low-grade DLS. The purpose of this study was to evaluate and compare the efficacy and safety betwwen MIS-TLIF and TO-TLIF in the treatment of single-level low-grade DLS.

The surgical plan was made mainly based on the precondition of patients’ general condition and preference, and each case was considered suitable for MIS or TO-TLIF. The surgeon's learning curve might affect the results^17^, all cases in our study were performed by the same surgeon who was well-experienced in MIS/TO-TLIF technique to avoid the bias of the operator. The MIS-TLIF technique directly reached the responsible segment through the Wiltse approach, and was performed with the assistance of minimally invasive devices without excessive dissection of the paravertebral muscles. Wang et al. proved that MIS-TLIF technique caused less paraspinal muscles injury than TO-TLIF through electrophysiological monitoring and MRI scanning^18^. Several studies had reported that MIS-TLIF technique was significantly better than TO-TLIF in terms of intraoperative bleeding, postoperative drainage, and length of hospital stay^19,20^. The results of this study regarding the above aspects were consistent with their findings. And some studies reported that the MIS-TLIF group had less postoperative blood transfusion^8,21^. Adogwa et al. observed that the dosage of postoperative analgesics in the MIS-TLIF group was significantly reduced^22^. Besides, our study found that the time of underground activities and return to work was earlier in patients experienced MIS-TLIF. The theoretical advantage of less soft tissue damage may be indirectly reflected in the fact that patients in the MIS-TLIF group returned to work earlier. But Liow, M. H. L considered that time taken to return to work did not influence outcomes of MIS-TLIF^23^. In addition, Parker compared the average hospitalization costs of patients and considered MIS-TLIF to be more economical^24^.

MIS-TLIF technique has reached a consensus on superiority in intraoperative bleeding and hospital stay, but controversy regarding operation time was exist. Some studies believe that there is no significant difference in the operation time between the two procedures^25,26^. Some studies reported that MIS-TLIF technique has a shorter operation time than TO-TLIF^19,20^. But some scholars considered that MIS-TLIF was associated with a longer operation time^27^. A meta-analysis of Qin et al. also proved that MIS-TLIF has a longer operation time^16^, and our research results are consistent with it. The dispute might be related to the learning curve of minimally invasive technology^28^. Compared with traditional surgery, the field of vision in minimally invasive surgery is relatively limited. MIS-TLIF surgery is similar to micro-carving, which operating in a limited space. It requires the surgeon's solid anatomical knowledge and long-term practical experience. In this study, there was no significant difference in terms of BMI and fusion segments, and the surgeon was well experienced with MIS technique. In addition, the surgeon in our study tended to restore the dislocated vertebral body under the limited field of vision and working space, which was reasonable to further explain why MIS-TLIF technique took longer time than open surgery.

Any technology has advantages and disadvantages, and MIS-TLIF is no exception. It relies on fluoroscopy to determine the anatomical position, which inevitably causes increased radiation exposure for patients and medical staff involved in surgery. Many studies reported MIS-TLIF had more fluoroscopy times than TO-TLIF when dealing with degenerative lumbar disease^21,29^. The systematic review of Phan et al. also confirmed the above results^8^, which were consistent with our study. In addition, we found that the perspective times was mainly concentrated in the screw insertion stage. Wang et al. considered that the perspective times were significantly reduced when the learning curve of MIS-TLIF technique reached the platform stage^26^. Besided, Kim et al. believe that MIS-TLIF technique with navigation assistance can significantly reduce radiation exposure^30^.

It is undeniable that the patient-reported outcome indicators (such as VAS and ODI) are of irreplaceable importance for the evaluation of clinical efficacy^30^. In our study, the postoperative VAS and ODI in each group were significantly improved compared with those before operation (*P* > 0.05). In addition, we divided the period of follow-up into two parts: short-term and mid-term. The short-term follow-up included 7 days, 3 months and 6 months, while the mid-term follow-up included 12 months and the last follow-up. A large number of studies have proved that MIS-TLIF had obvious benefits in VAS and ODI scores of short-term follow-up^5,31,32^, and our results were similar to them. It's well understood that open TLIF technique involves more soft tissue stripping, which fully reflects the advantages of MIS-TLIF. As mentioned before, MIS-TLIF technique has less damage to soft tissue, less intraoperative bleeding, and faster postoperative recovery. Although the short-term efficacy had reached consensus, the mid/long-term effect is controversial. Several studies reported that MIS-TLIF was superior to TO-TLIF in VAS and ODI scores at more than 1 year of follow-up^33,34^. The study of systematic review also agreed with that^8^. But Lin’s systematic review found that although MIS-TLIF had a slight advantage in VAS of mid/long-term follow-up, the results were similar in ODI score^35^. According to the latest meta-analysis, there was no significant difference between the two techniques in the mid/long-term VAS score, but MIS-TLIF has obvious advantages in ODI score^15^. However, due to the lack of high-quality studies, the conclusions of these meta-analyses were weak. Wang et al. reported that no significant difference was observed between the two techniques in the mid/long-term VAS and ODI scores^26^, which was recognized by some scholars^36–38^. And our study also found that the VAS and ODI scores in the two groups were similar at 12 months and the last follow-up, which indicated that there was no significant difference between MIS-TLIF and TO-TLIF in the mid-term clinical efficacy, but further study of longer-term follow-up is needed for verification. Why was the clinical efficacy similar in mid/long-term clinical efficacy? Kaloostian considered that it might be related to the self-healing and compensatory effect of human body^39^. Traditional open TLIF technique did cause great damage to soft tissue, but patients would reach the same point at which the patients experienced MIS-TLIF had been a few weeks or more earlier. Despite the controversy, all the aforementioned literature considered that VAS and ODI scores at mid/long-term follow-up in MIS-TLIF group were at least as good as, if not better than, those in TO-TLIF group.

At present, for low-grade degenerative lumbar spondylolisthesis, whether there is a need for forced reduction during surgery is not clear. Gong believed that the intraoperative reduction in TO-TLIF technique did not lead to better clinical efficacy in the treatment of low-grade DLS^40^. In clinical efficacy of patients experienced MIS-TLIF technique, Fan also found that no significant difference was observed between intraoperative reduction group and in situ fusion group^41^. In this study, the surgeon preferred intraoperative reduction, as which seems theoretically attractive. Also, reduction can better restore the spine to the anatomical position and increase the area of intervertebral bone grafting. Besides, Kida et al. considered that intraoperative reduction can relieve early muscle fatigue and back pain which caused by low lordosis, and prevent degeneration of adjacent intervertebral discs^42^. However, the above opinions had no evidence-based proof. In our study, significant improvement in vertebral slip ratio was observed at time-point of immediately after surgery and last follow-up when comparing with preoperative status in each group(*P* < 0.05). And no significant difference was exist in vertebral slip ratio between the status of last follow-up and immediately after surgery in each group (*P* > 0.05). Moreover, no statistical difference was detected in vertebral slip ratio at any time-point of follow-up between the MIS-TLIF group and TO-TLIF group (*P* > 0.05). It showed that both groups had achieved similar good results in reduction.

Complications can be used to evaluate the security of a technology^43^. There were no deep infections in both groups in this study. One case of superficial incision infection occurred in the MIS-TLIF group, and two cases appeared in the TO-TLIF group. The three patients were all cured by strengthening dressing change and anti-infection treatment. MIS-TLIF technique has the advantages of small incisions, but excessive pursuit of small incisions will inevitably require excessive expansion of the surgical channel, which can easily lead to ischemia due to pressure on the skin margin. Some scholars considered that MIS-TLIF had more complications such as tear of the dural sac and cerebrospinal fluid leakage during the initial development^44^. The surgeon in this study had extensive experience with MIS-TLIF technique, and there was no tearing of the dural sac during the operation. One case of postoperative cerebrospinal fluid leakage occurred in the TO-TLIF group, which was not found during surgery. The case was placed in a head-low position and treated by fluid replacement and preventing infection. After delayed extubation and intermittent clamping treatment, it improved within 1 week and no obvious neurological deficit occurred. In both groups of this study, neither screw loosening nor cage displacement was found, and complete interbody fusion was observed in all case at last follow-up. Park considered that successful intervertebral fusion provided the basis for a good clinical outcome^45^, while Lamberg believed that there was no inevitable relationship between fusion and clinical efficacy^46^.

Finally, it is necessary to point out the limitations of this study. First, it was a retrospective comparative study, which might have a potential methodological defects and risks of bias; second, no data was collected on segmental lordosis and intervertebral foramen height; third, correlation analysis was not performed beween radiological evaluation and clinical effect; fourth, the sample size in each arm of this study was too small, which might affect the results. In the next step, we will conduct a prospective, large sample, more comparative indicators, and longer-term follow-up study.

## Conclusion

MIS-TLIF and TO-TLIF both achieve satisfactory clinical efficacy in the treatment of low-grade single-segment LDS. The MIS technology showed better short-term improvement in BP-VAS and ODI, less blood loss, shorter hospital stay, and quicker return to work than TO-TLIF, however there was more radiation exposure and no difference in complications or radiographic outcomes.
